# Supplementary material for: MetaQTL: a package of new computational methods for the meta-analysis of QTL mapping experiments
Source: BMC Bioinformatics. 2007 Feb 8;8:49. doi: 10.1186/1471-2105-8-49 (PMC1808479; doi:10.1186/1471-2105-8-49)
Supplement: Additional File 7 — MetaQTL Package : jar file and tutorial. This Zip archive contains both the MetaQTL JAR file and the files of the tutorial. [file 1471-2105-8-49-S7.zip › org.inra.metaqtl/doc/org/thalia/metaqtl/main/MetaSimuStat.html]

MetaSimuStat


|  |  |  |
| --- | --- | --- |
| **Overview** \| **Package** \| **Class** \| **Use** \| **Tree** \| **Deprecated** \| **Index** \| **Help** | |  |
| **PREV CLASS**   NEXT CLASS | **FRAMES**    **NO FRAMES**     **All Classes** |  |
| SUMMARY: NESTED \| FIELD \| CONSTR \| METHOD | DETAIL: FIELD \| CONSTR \| METHOD |  |


---


## org.thalia.metaqtl.main Class MetaSimuStat

```
java.lang.Object
  MetaMain
      org.thalia.metaqtl.main.MetaSimuStat
```

---

``` public class MetaSimuStat extends MetaMain ```

---

**Constructor Summary**

`MetaSimuStat()`


| **Method Summary** | |
| --- | --- |
| `static int[]` | `getK(org.thalia.metaqtl.main.MetaSimuStat.SimuConfig[] results, java.lang.String name)` |
| `static double[]` | `getMu(org.thalia.metaqtl.main.MetaSimuStat.SimuConfig[] results)` |
| `static int[]` | `getO(org.thalia.metaqtl.main.MetaSimuStat.SimuConfig[] results)` |
| `static double[]` | `getPredict(org.thalia.metaqtl.main.MetaSimuStat.SimuConfig[] results, java.lang.String name)` |
| `static double[]` | `getSD1(org.thalia.metaqtl.main.MetaSimuStat.SimuConfig[] results)` |
| `static double[]` | `getSD2(org.thalia.metaqtl.main.MetaSimuStat.SimuConfig[] results)` |
| `static double[]` | `getX(org.thalia.metaqtl.main.MetaSimuStat.SimuConfig[] results)` |
| `static void` | `main(java.lang.String[] args)` |
| `void` | `printHelp()` |
| `void` | `printUsage()` |

**Methods inherited from class java.lang.Object**

`equals, getClass, hashCode, notify, notifyAll, toString, wait, wait, wait`

**Constructor Detail**

### MetaSimuStat

```
public MetaSimuStat()
```


**Method Detail**

### printUsage

```
public void printUsage()
```

---


### printHelp

```
public void printHelp()
```

---


### main

```
public static void main(java.lang.String[] args)
```

---


### getMu

```
public static double[] getMu(org.thalia.metaqtl.main.MetaSimuStat.SimuConfig[] results)
```

---


### getX

```
public static double[] getX(org.thalia.metaqtl.main.MetaSimuStat.SimuConfig[] results)
```

---


### getO

```
public static int[] getO(org.thalia.metaqtl.main.MetaSimuStat.SimuConfig[] results)
```

---


### getSD1

```
public static double[] getSD1(org.thalia.metaqtl.main.MetaSimuStat.SimuConfig[] results)
```

---


### getSD2

```
public static double[] getSD2(org.thalia.metaqtl.main.MetaSimuStat.SimuConfig[] results)
```

---


### getK

```
public static int[] getK(org.thalia.metaqtl.main.MetaSimuStat.SimuConfig[] results,
                         java.lang.String name)
```

---


### getPredict

```
public static double[] getPredict(org.thalia.metaqtl.main.MetaSimuStat.SimuConfig[] results,
                                  java.lang.String name)
```


---


|  |  |  |
| --- | --- | --- |
| **Overview** \| **Package** \| **Class** \| **Use** \| **Tree** \| **Deprecated** \| **Index** \| **Help** | |  |
| **PREV CLASS**   NEXT CLASS | **FRAMES**    **NO FRAMES**     **All Classes** |  |
| SUMMARY: NESTED \| FIELD \| CONSTR \| METHOD | DETAIL: FIELD \| CONSTR \| METHOD |  |


---
